# Improving dynamic balance in relapsing-remitting MS: insights from robotic-assisted rehabilitation therapy

**DOI:** 10.1186/s12984-025-01856-w

**Published:** 2026-01-09

**Authors:** Jessica Podda, Ludovico Pedullà, Giorgia Marchesi, Valentina Squeri, Alice De Luca, Alice Bellosta, Giulia Rogina, Andrea Vitiello, Laura Isolabella, Margherita Monti Bragadin, Mario Alberto Battaglia, Andrea Tacchino, Giampaolo Brichetto

**Affiliations:** 1 NeuroBRITE Research Center, Italian Multiple Sclerosis Foundation, Genoa, Italy; 2 Movendo Technology s.r.l, Genoa, Italy; 3 Italian Institute of Technology, Genoa, Italy; 4 Department of Neuroscience, Rehabilitation, Ophthalmology, Genetics, Maternal and Child Health, University of Genoa, Genoa, Italy; 5 Italian Multiple Sclerosis Society, Genoa, Italy; 6 Department of Life Science, University of Siena, Siena, Italy; 7 Italian Multiple Sclerosis Foundation, Genoa, Italy

## Abstract

**Background:**

Balance deficits affect over 75% of people with multiple sclerosis (PwMS), significantly limiting mobility and daily activities. To address these symptoms, technology-assisted rehabilitation has recently shown promise for restoring balance. This study aimed to test the non-inferiority of a rehabilitative protocol that combines robotic-assisted training with conventional physiotherapy (ROB) compared to a protocol consisting solely of traditional balance training (TRAD) for PwMS.

**Methods:**

Forty-two PwMS with relapsing-remitting course were randomly assigned to either ROB or TRAD group. Participants had to undergo 20 sessions (2/week for 10 weeks, 45 min each), focusing on balance exercises targeting static and dynamic control. In the ROB group, traditional exercises were progressively integrated with robotic-based tasks using the hunova^®^ system (Movendo Technology s.r.l., Genoa, Italy). The Berg Balance Scale (BBS) was the primary outcome measure, while the Two-Minute Walking Test (2MWT) and the Composite Score (COMP) from the Sensory Organization Test were selected as secondary outcomes. Additional robotic metrics from hunova^®^ were analyzed to assess stability under static and dynamic conditions.

**Results:**

Both groups showed significant overall improvements in BBS (*p* = 0.003), 2MWT (*p* = 0.031), and COMP (*p* < 0.001), supporting the non-inferiority of the robotic-assisted protocol. However, participants in the ROB group demonstrated additional improvements in unstable conditions compared to TRAD group. Specifically, elastic balance tasks resulted in significant reductions in path length (*p* = 0.047) and trunk variability (*p* = 0.017). Additionally, reactive balance metrics showed significant decreases in the first oscillation time for both left and right directions (*p* = 0.027 and *p* = 0.029), as well as in the average oscillation time for both directions (*p* = 0.006 and *p* = 0.012).

**Conclusions:**

Robotic-assisted rehabilitation combined with conventional physiotherapy is at least as effective as traditional therapy for improving balance in PwMS, demonstrating non-inferiority for the primary outcome. Additionally, the greater dynamic balance improvements observed in the ROB group suggest that robotic technology may provide added benefits by enhancing specific balance mechanisms. Since these improvements persisted for at least two months post-treatment, robotic-assisted training may serve as a complementary or alternative approach to conventional rehabilitation strategies for balance disorders in MS.

**Supplementary Information:**

The online version contains supplementary material available at 10.1186/s12984-025-01856-w.

## Introduction

Multiple sclerosis (MS) is the most prevalent chronic inflammatory disease of the central nervous system (CNS), affecting > 2 million people worldwide and currently incurable [1]. Its clinical course is unpredictable, with highly variable manifestations [2]. Among the most disabling symptoms, balance impairment is a major issue in MS leading to an increased risk of falling [3]. This often prevents people with MS (PwMS) from performing their daily living activities [4, 5], negatively impacting their quality of life [6]. Compared to healthy controls of similar age, PwMS experience more falls as a result of these impairments [7]. Indeed, a meta-analysis on falls in MS showed that 56% of PwMS fall at least once each 3 months [8].

CNS damage in MS disrupts the integration of sensory signals from multiple sources, as muscle, tendon, joint proprioceptors, skin exteroceptors, vestibular, and visual inputs, resulting in impaired postural responses needed to maintain balance [9]. Targeted interventions that enhance postural reactions in both stable and unstable conditions can improve balance and gait, potentially leading to greater activity participation and quality of life in PwMS, regardless of disability severity [10–12]. To this aim, sensory-integration strategies have shown promise in addressing these challenges [9, 13–15]. People with difficulties in the organization and integration of information from multiple sensory systems could have problems especially in safely navigating through environments, with conflicting visual surroundings information, surface changes/irregularities, or obstacles on the ground [16]. Perturbation-based balance training, which involves exposing individuals to controlled disturbances to enhance their neuromuscular responses, has been shown to improve reactive balance control and reduce fall rates [17]. In this context, technology-aided rehabilitation is an emerging approach that offers great potential for restoring motor functions and/or enhancing sensory-motor integration strategies, thanks to sophisticated electronic components and novel computational methods in both PwMS and other populations with neurological disorders. Some new technologies have been advocated to help therapists delivering a repeated, intensive and standardized practice of skilled motor tasks in a motivating, engaging, and enriched environment while addressing movement quality. Current evidence suggests that robotic-assisted rehabilitation treatment should complement conventional physical therapy to maximize its effectiveness in people with neurological diseases [18, 19]. Studies have shown that robotic devices can be valuable in supporting physical therapy, improving outcomes such as motor control and balance when used alongside conventional methods, although the impact of treatment dose and duration still remains uncertain for PwMS [19, 20].

Based on this, the aim of our study was to test whether a rehabilitative protocol that combines robotic-assisted training with conventional physiotherapy (ROB) is at least non-inferior to a protocol consisting solely of traditional balance training (TRAD) for PwMS. Specifically, we hypothesized that: (1) the ROB protocol would be at least non-inferior to TRAD on standard clinical measures of balance and walking ability; (2) the ROB protocol might produce additional improvements in robot-specific, fine-grained balance measures, reflecting potential benefits of robotic-assisted training that are difficult to capture with conventional clinical tests. The rationale behind the study is twofold. First, it would explore the potential field of application of robotic solutions, which is increasingly being used in clinical practice even though their mechanisms of action are still unknown. Second, it would be relevant to find novel approaches which allow training PwMS in a safe and efficient manner.

## Materials and methods

### Participants

PwMS were recruited among those followed as outpatients at the AISM Rehabilitation Service of Genoa (Italy). Inclusion criteria were: MS diagnosis according to revised McDonald criteria [21], relapsing-remitting (RR) course, age between 18 and 75 years, disability level as measured by the Expanded Disability Status Scale (EDSS) [22] ≤ 6, stable phase of disease without relapses or worsening in the last three months, balance as measured by the Berg Balance Score (BBS) [23] > 35, indicating ability to stand upright and walking with at least one support, and normal cognitive functioning as indicated by a Montreal Cognitive Assessment (MoCA) [24, 25] score ≥ 24 and by the Symbol Digit Modalities Test (SDMT) cut-off ≥ 34.2 [26]. Exclusion criteria were: psychiatric disorders, significant visual impairment defined as a Visual System scoring more than 2 at the Functional Systems Score of EDSS and cardiovascular and/or respiratory disorders. All study procedures and consent forms conformed to the ethical standards of the 2013 revised Declaration of Helsinki and were approved by the regional ethical committee (Comitato Etico Regionale Liguria, reference number: 36/2022 - DB id 12144, Italy). The participants provided informed consent to participate in the study and to the publication of the results.

### Rehabilitation intervention

PwMS who met all eligibility criteria were randomly assigned to one of the two intervention groups based on a random sorting procedure using a computer-generated sequence: (1) ROB (robotic training integrated with conventional therapy); (2) TRAD (traditional physiotherapy). Both the ROB and TRAD protocols were developed in close collaboration with the physiotherapists. Given the novelty of the robotic assisted device used in this study, after a specific training, they tested all the exercises and identified those most suitable for PwMS, following a co-creation approach with the research team. All PwMS, regardless of the randomization group, underwent treatments of equal duration and functional intensity, designed to improve static and dynamic balance, both with open and closed eyes. Participants in both groups had to undergo 20 sessions (2 sessions/week, for a total of 10 weeks) of 45 min each. Training was composed of four phases of five sessions, each incorporating exercises with increasing functional demands and levels of difficulty: Phase (1) basic postural control and static balance, passive mobilization, and initial reaching tasks; Phase (2) elastic balance training and elastic movements to enhance dynamic stability; Phase (3) impulsive balance tasks, proprioceptive exercises, and reaching tasks under varied conditions; Phase (4) advanced balance control, incorporating monopodal stance exercises and fluid dynamic balance tasks to simulate real-life demands. Exercises were progressively adapted in complexity and difficulty based on individual performance, with participants either progressing to the next phase or remaining in the same phase according to the physiotherapist’s clinical judgment.

**Fig. 1:**
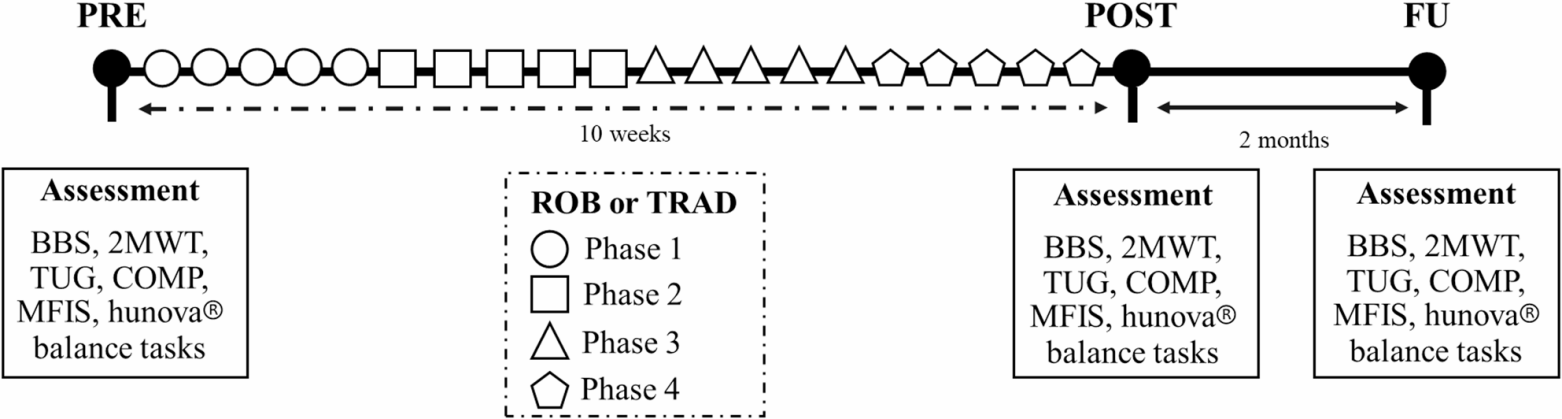
Experimental procedure timeline. Each evaluation consisted of both clinical and robot-based assessments. The experiment started with the first evaluation (PRE), followed by 10 weeks of rehabilitation, which included two types of interventions: robot-assisted rehabilitation integrated with conventional physiotherapy (ROB) and traditional rehabilitation (TRAD). A second evaluation (POST) was conducted at the end of the treatment period. Finally, a follow-up (FU) assessment took place two months after the POST evaluation

See Fig. 1 for an overview of the experimental procedure. In each phase, each training session followed a specific structure. All subjects started with 15 min of treadmill training. Participants walked on a treadmill at a self-selected comfortable speed, with supervision by a physiotherapist to ensure safety and proper gait mechanics. Speed was gradually adjusted according to the participant’s tolerance and functional ability. Then the other 30 min differed for the two groups.

Specifically, TRAD intervention consisted of 30 min of static and dynamic balance exercises. This included progressively challenging activities aimed at improving postural control, weight shifting, and lower-limb stability. Exercises comprised single-leg and double-leg stance tasks, half-kneeling exercises, and core stabilization such as bridging with a foam under the feet, “Superman” pose in quadruped, tandem stance tasks, single- and double-leg stance, half-kneeling and core stabilization using Bobath balls [9]. Difficulty was systematically increased by modifying base of support, visual input, and task complexity, allowing participants to train balance under progressively more demanding conditions.

ROB intervention consisted of static and dynamic exercises, similar to those in the TRAD group and balance training on the hunova^®^ platform (Movendo Technology s.r.l., Genoa, Italy), initially seated and progressing to bipedal and single-leg standing positions. Participants performed a variety of balance and postural tasks, reaching point-to-point with trunk or upper limbs, drawing patterns with the trunk, reactive perturbations, and load shifting on one leg at different percentages (e.g., 30%, 50%, 70% of body weight), while the platform was stable and unstable. The duration of robotic balance training increased progressively from 15 to a maximum of 30 min, while traditional static and dynamic exercises were gradually reduced. The progressive integration was planned so that exercises delivered via the hunova^®^ gradually replaced corresponding traditional exercises, as determined during the protocol development in consultation with physiotherapists. In the ROB condition, the exact exercise duration and progression were strictly controlled by the hunova^®^ platform, ensuring precise timing and standardization. In contrast, the TRAD sessions followed standard clinical practice, where physiotherapists adjusted exercises according to participant needs and tolerance; session time was therefore consistent overall but not enforced at the same level of precision as in ROB.

hunova^®^ is an advanced robot-assistive device designed for lower limb, pelvis, core, and trunk assessment and rehabilitation across various clinical settings (neurology, orthopaedics, geriatrics) [18, 27–29]. This device enables the evaluation of balance while standing (both in mono- and bi-podalic configurations) and sitting, both in static and dynamic conditions; it has different difficulty levels for each task, and it integrates both visual and auditory feedback based on the user’s performance. The device has been already validated in PwMS for static and dynamic balance assessment [16, 30]. hunova^®^ consists of two electromechanical and sensorized platforms with two degrees of freedom (forwards/backwards and left/right), one at the foot level and one at the seat level. Behind each platform, a six-axis force-torque sensor allows the estimation of the center of pressure, while an optical incremental encoder allows the measurement of the inclination of the platforms. The device integrates an Inertial Measurement Unit (IMU) synchronized via software, placed on the sternum of the user to monitor trunk motion, as previously validated [31]. hunova^®^ also comprises a tablet device which runs the clinician user interface. This graphical user interface (GUI) allows the operator to manage patients’ database, single patient’s training and assessment history and start exercise sessions directly from the tablet. The tablet is connected to the robotic system via a WiFi link.

Each session was personalized according to the clinical characteristics of the participants, as well as to minimize boredom or frustration: exercise parameters were set in line with each subject’s impairment and their performance during training, to match their specific needs and provide a training proportional to their capabilities [32], but also sufficiently challenging (i.e., neither too easy nor too difficult). To reduce confounding effects, participants were restricted from engaging in additional physical therapy (e.g., functional rehabilitation therapy, aquatic therapy, postural therapy such as the Mézières method) during the 10-week intervention and for two months post-treatment. However, other therapies (psychological therapy, speech therapy, occupational therapy, manual therapy, pelvic floor rehabilitation) were allowed, if clinically indicated.

### Assessment

PwMS were tested at the beginning of treatment (PRE), at the end of treatment (POST), and after two months from POST (FU). The participants were administered several clinical tests for balance and walking. The Berg Balance Scale (BBS), a validated and reliable scale to assess balance in MS ^33^, has been selected as the primary outcome, while the Composite score (COMP) from the Sensory Organization Test (SOT) [34] of Equitest (NeuroCom International, Inc., Clackamas, OR), the Timed-up-and-go-test (TUG) [33], and the Two-Minute Walk Test (2MWT) [35] have been selected as secondary outcomes.

Balance was evaluated also with hunova^®^ under different conditions:

- Balance on static platform (Static Balance) that tests the ability to maintain the position of the center of mass in unsupported stand when the base of support does not change, in both open eyes (EO) and closed eyes (EC) conditions;
- Balance on dynamic unstable platform (Elastic Balance) that estimates the subject’s stability in a non-static situation in which the movement of the platform depends on the subject’s movement, in both EO and EC conditions;
- Balance on dynamic perturbating platform (Reactive Balance) that evaluates the reactive postural control component of balance, defined as the ability to recover stability after an external random perturbation in different directions (forward, left, right), only in EO condition;
- Stability limits (Stability Limits) that measures the maximum voluntary posture reached in the absence of external perturbations, only in EO condition.

Table 1 presents a list of the Center of pressure (CoP) parameters computed by hunova^®^ grouped for different balance tasks. All these indicators are proportional to the instability of the subjects: the greater the values, the lesser the subject’s ability to maintain balance.

**Table 1:** List of the parameters computed by hunova^®^

Parameter (abbreviation)	hunova^®^ task	Description
Sway Area (SA)	Static Balance	The area of the 95% confidence ellipse of the statokinesigram of the trunk accelerations in the horizontal plane (expressed in cm2).
Max oscillation range in medio-lateral orientation (MLO range)	Static Balance	The extent of oscillations in the ML direction, computed by looking at the maximum and minimum shift of the CoP coordinates in the two principal directions (expressed in cm).
Max oscillation range in antero-posterior orientation (APO range)	Static Balance	The extent of oscillations in the AP direction, computed by looking at the maximum and minimum shift of the CoP coordinates in the two principal directions (expressed in cm).
Path Length (Path_lenght)	Static Balance	The total length of the trajectory of the CoP (expressed in cm).
Romberg	Static Balance	The ratio between the open-eye area and the closed-eye area.
Sway Area (SA)	Elastic Balance	The area of the 95% confidence ellipse of the statokinesigram of the trunk accelerations in the horizontal plane (expressed in cm2).
Max oscillation range in medio-lateral orientation (MLO range)	Elastic Balance	The extent of oscillations in the ML direction, computed by looking at the maximum and minimum shift of the CoP coordinates in the two principal directions (expressed in cm).
Max oscillation range in antero-posterior orientation (APO range)	Elastic Balance	The extent of oscillations in the AP direction, computed by looking at the maximum and minimum shift of the CoP coordinates in the two principal directions (expressed in cm).
Path Length (Path_lenght)	Elastic Balance	The total length of the trajectory of the CoP (expressed in cm).
Trunk Variability (Trunk_var)	Elastic Balance	The variability of the oscillations of torso around the initial position, measured as the SD of the accelerations by the sensor place on the torso.
First oscillation time in Frontal direction (First_osc_time_front)	Reactive Balance	The average time it takes for the patient’s torso to stabilize after the first oscillation in frontal direction (expressed in sec).
First oscillation time in Left direction (First_osc_time_left)	Reactive Balance	The average time it takes for the patient’s torso to stabilize after the first oscillation in left direction (expressed in sec).
First oscillation time in Right direction (First_osc_time_right)	Reactive Balance	The average time it takes for the patient’s torso to stabilize after the first oscillation in right direction (expressed in sec).
Oscillation time in Frontal direction (Osc_time_front)	Reactive Balance	The average time it takes for the patient’s torso to stabilize in frontal direction (expressed in sec).
Oscillation time in Left direction (Osc_time_left)	Reactive Balance	The average time it takes for the patient’s torso to stabilize in left direction (expressed in sec).
Oscillation time in Right direction (Osc_time_right)	Reactive Balance	The average time it takes for the patient’s torso to stabilize in right direction (expressed in sec).
Sway Area (SA)	Stability Limits	The area of the 95% confidence ellipse of the statokinesigram of the trunk accelerations in the horizontal plane (expressed in cm2).
Max CoP in back direction (Max_cop_back)	Stability Limits	The maximum movement of the CoP or the area covered by the stability cone in back direction (expressed in cm).
Max CoP in frontal direction (Max_cop_front)	Stability Limits	The maximum movement of the CoP or the area covered by the stability cone in frontal direction (expressed in cm).
Max CoP in right direction (Max_cop_right)	Stability Limits	The maximum movement of the CoP or the area covered by the stability cone in right direction (expressed in cm).
Max CoP in left direction (Max_cop_left)	Stability Limits	The maximum movement of the CoP or the area covered by the stability cone in left direction (expressed in cm).

Additionally, PwMS were required to fulfil the Modified Fatigue Impact Scale (MFIS) [36] in order to gather information about perceived fatigue.

### Statistical analysis

Sample size was determined by comparing means and changes in the primary outcomes (i.e., BBS score) following previous results [13]. The criterion for significance (α) was set at 0.05 (two-tailed) and the statistical power at least 80%. Considering a possible drop-out of 10%, the proposed sample size was of 44 (22 participants for both groups).

Main descriptive statistics (mean, standard deviation, standard error) were used to analyze sample clinical characteristics. Independent t-tests were run to investigate any difference across groups at baseline considering demographic, clinical and traditional balance and walking measures.

A repeated-measures ANOVA analysis (RM-ANOVA) on the primary and secondary outcomes, both computed by traditional tests and extracted by hunova^®^ (see Table 1), was carried out with groups (ROB, TRAD) as between subjects-factor and time (PRE, POST e FU) as within subjects-factor to investigate any differences across groups at different time points.

Since static balance test represents the reference condition, a RM-ANOVA on static balance outcomes from hunova^®^ was further carried out with condition (EO, EC) and time (PRE, POST, FU) as within subjects-factors and group (ROB, TRAD) as between subjects-factor. Normally in this condition, it is expected that without visual information there will be slightly larger oscillations of the COP [37]. The significance level was set to *p* ≤ 0.05. Statistical analysis was performed utilizing SPSS for Windows.

## Results

### Demographic and clinical variables

Overall, 44 PwMS were eligible, enrolled and then randomly assigned to the ROB and TRAD groups. Two participants in TRAD abandoned the study before POST evaluation due to personal problems. Therefore, forty-two PwMS (32 females) completed the study (N ROB = 22; N TRAD = 20). No differences between groups were present at baseline in demographic and clinical variables as age (ROB = 55.09 ± 10.36; TRAD = 51.90 ± 8.56; *p* = 0.286), EDSS (ROB = 3.70 ± 1.36; TRAD = 4.17 ± 1.39; *p* = 0.276), disease duration (ROB = 12.63 ± 8.40; TRAD = 14.15 ± 10.53; *p* = 0.608) and BBS (ROB = 49.28 ± 4.89; TRAD = 48.40 ± 5.72; *p* = 0.592). No difference between groups in the number of treatment session was found (ROB = 17.00 ± 1.85; TRAD = 15.75 ± 3.75; *p* = 0.173). PwMS did not report any falls during or out of the training sessions and no other adverse events were observed during the study period. All results from independent t-test are presented in Table 2.

**Table 2:** Sample demographic and clinical characteristics at PRE

Variable	Group	*N*	Mean	SD	*p*
Age (years)	ROB	22	55.09	10.36	0.286
	TRAD	20	51.90	8.56	
EDSS (score)	ROB	22	3.70	1.36	0.276
	TRAD	20	4.17	1.40	
Disease duration (years)	ROB	22	12.63	8.40	0.608
	TRAD	20	14.15	10.53	
BBS (score)	ROB	22	49.28	4.89	0.322
	TRAD	20	48.40	5.72	
2MWT (m)	ROB	22	99.19	22.20	0.592
	TRAD	20	90.95	30.71	
TUG (sec)	ROB	22	11.30	5.54	0.936
	TRAD	20	11.43	5.07	
COMP (SOT) (score)	ROB	22	67.33	13.28	0.972
	TRAD	20	67.17	14.91	
MFIS_ PHYS (score)	ROB	22	20.91	6.32	0.383
	TRAD	20	18.55	10.64	
MFIS_COG (score)	ROB	22	17.09	9.73	0.130
	TRAD	20	12.50	9.473	
MFIS_PSY-SOC (score)	ROB	22	3.82	1.71	0.208
	TRAD	20	3.00	2.41	
MFIS_TOT (score)	ROB	22	41.82	13.98	0.141
	TRAD	20	34.05	19.37	
Treatment session number (N)	ROB	22	17.00	1.85	0.173
	TRAD	20	15.75	3.75	

### Balance outcomes from traditional scales and tests

**Fig. 2:**
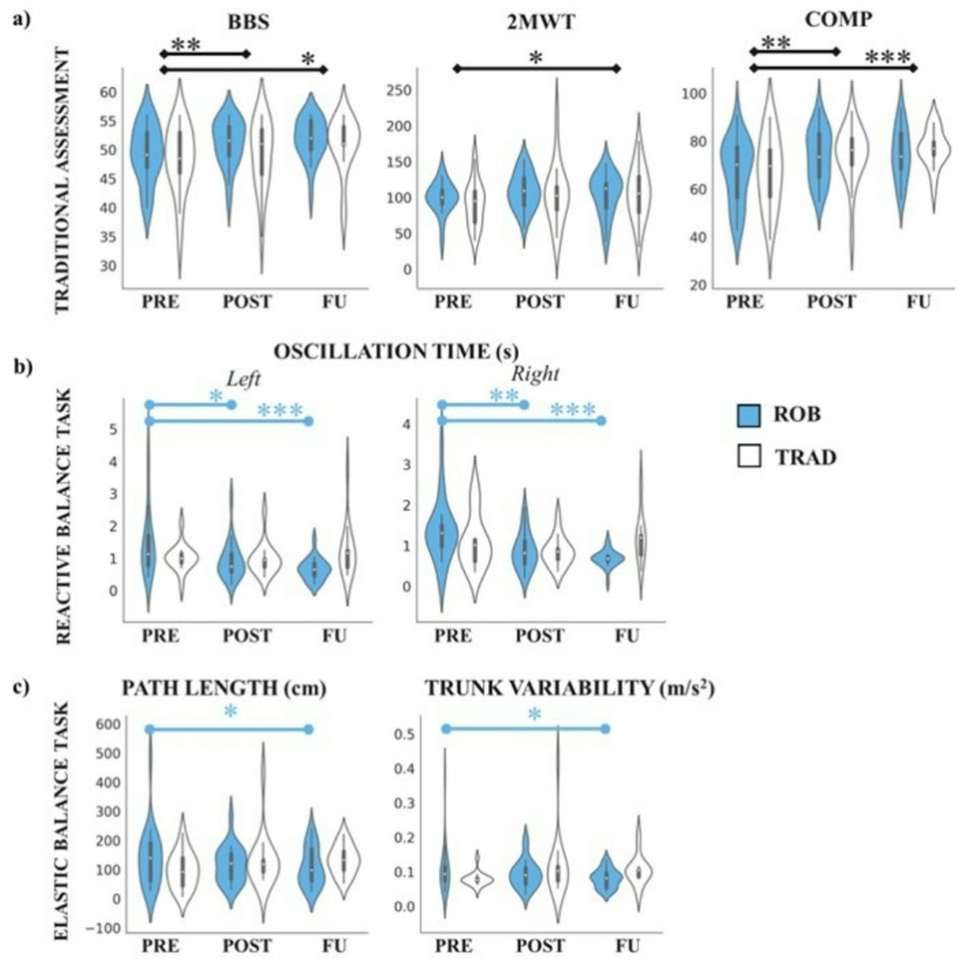
Results from RM ANOVA on selected outcome measures. **A** Displays findings from RM ANOVA on BBS, 2MWT and COMP. **B** presents total oscillation time in the reactive balance, while **C** shows path length and trunk variability in the EO condition in the elastic balance. ROB is represented in blue, and TRAD in white. For **B** and **C**, significant time*group post-hocs are highlighted in blue (indicating significant post-hoc results for ROB). Asterisks denote significance levels: * *p* < 0.05, ** *p* < 0.010, *** *p* < 0.001

RM-ANOVA on clinical outcomes revealed a main effect of time on the primary outcome BBS, demonstrating that participants improved over time independently from the type of intervention (Mean ± Standard Error: PRE: 48.84 ± 0.82; POST: 50.44 ± 0.75; FU: 51.36 ± 0.65; *p* = 0.003); the post-hoc analyses revealed a significant difference between PRE and POST (*p* = 0.008) and PRE and FU (*p* = 0.017). In addition, a main effect of time on 2MWT (PRE: 95.07 ± 4.11; POST: 106.30 ± 5.31; FU: 104.83 ± 5.07; *p* = 0.031) and COMP (PRE: 67.25 ± 2.17; POST: 74.04 ± 1.75; FU: 75.63 ± 1.48; *p* < 0.001) was observed; the post-hoc analysis revealed significant differences between PRE and POST (*p* = 0.020) for 2MWT, and between PRE and POST (*p* = 0.005) and PRE and FU (*p* < 0.001) for COMP. No other significant effects were observed for TUG (PRE: 11.36 ± 0.82; POST: 10.52 ± 0.62; FU: 11.51 ± 0.82; *p* = 0.201) and MFIS total score (PRE: 37.93 ± 0.2.59; POST: 35.67 ± 2.69; FU: 33.63 ± 2.45; *p* = 0.077). See Table 3 for a detailed description of the results from RM-ANOVA. See Fig. 2A for a representation of results for significant main effects.

**Table 3:** Results from RM ANOVA on balance, walking and fatigue variables from traditional scales and tests

Parameter	Group	Time	Mean	SE	RM ANOVA, *p*	
BBS	ROB	PRE	49.29	1.13	*TIME*, *p* = 0.003	
		POST	51.20	1.04	TIME * GROUP, *p* = 0.952	
		FU	51.86	0.89		
	TRAD	PRE	48.40	1.19		
		POST	49.68	1.09		
		FU	50.87	0.94		
2MTWT	ROB	PRE	99.19	5.67	*TIME*, *p* = 0.031	
		POST	108.29	7.33	TIME * GROUP, *p* = 0.261	
		FU	104.14	7.00		
	TRAD	PRE	90.95	5.94		
		POST	104.32	7.69		
		FU	105.53	7.34		
TUG	ROB	PRE	11.30	1.13	TIME, *p* = 0.201	
		POST	9.80	0.85	TIME * GROUP, *p* = 0.051	
		FU	10.18	1.13		
	TRAD	PRE	11.43	1.19		
		POST	11.24	0.90		
		FU	12.84	1.19		
COMP	ROB	PRE	67.33	3.00	*TIME*, *p* < 0.001	
		POST	73.79	2.42	TIME * GROUP, *p* = 0.567	
		FU	74.44	2.04		
	TRAD	PRE	67.17	3.15		
		POST	74.30	2.54		
		FU	76.81	2.14		
MFIS_ PHYS	ROB	PRE	20.91	1.84	TIME, *p* = 0.177	
		POST	19.91	1.82	TIME * GROUP, *p* = 0.440	
		FU	19.95	1.74		
	TRAD	PRE	18.55	1.93		
		POST	18.10	1.91		
		FU	15.62	1.82		
MFIS_COG	ROB	PRE	17.09	2.05	TIME, *p* = 0.107	
		POST	16.09	2.05	TIME * GROUP, *p* = 0.167	
		FU	16.62	1.86		
	TRAD	PRE	12.50	2.15		
		POST	11.35	2.15		
		FU	9.12	1.95		
MFIS_PSY-SOC	ROB	PRE	3.82	0.44	TIME, *p* = 0.158	
		POST	3.09	0.44	TIME * GROUP, *p* = 0.533	
		FU	3.57	0.46		
	TRAD	PRE	3.00	0.46		
		POST	2.80	0.46		
		FU	2.37	0.48		
MFIS_TOT	ROB	PRE	41.82	3.57	TIME, *p* = 0.077	
		POST	39.09	3.71	TIME * GROUP, *p* = 0.249	
		FU	40.14	3.39		
	TRAD	PRE	34.05	3.75		
		POST	32.25	3.89		
		FU	27.12	3.55		

### Balance outcomes from hunova^®^

Four participants in TRAD group missed one of the three robotic evaluations on hunova^®^ (i.e., static balance, elastic balance, reactive balance and stability limits) due to personal reasons so they have been excluded from these analyses (ROB *N* = 22; TRAD *N* = 16).

*Static balance.* Results from RM-ANOVA with condition (EO, EC) and time (PRE, POST, FU) as within subjects factor and group (ROB, TRAD) as between subjects factor showed a main effect of condition, confirming that EO condition was performed significantly better than EC condition for all variables considered (all ps < 0.001) [30, 38]. However, when evaluating differences across time and groups, no significant main effect of time or time*group interaction were found across variables in static balance task (all ps > 0.05) (see Table 1 in Additional file 1).

**Fig. 3:**
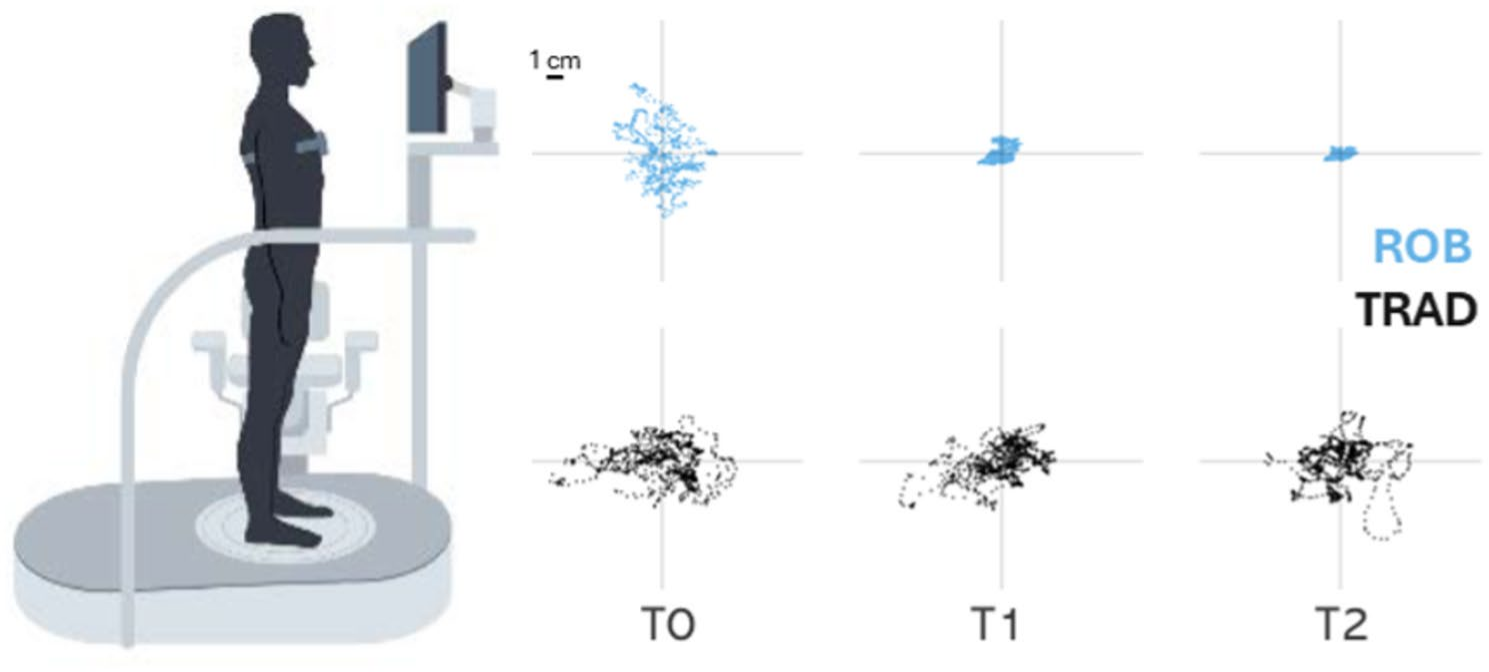
Statokinesiogram of the CoP sway path in EO condition during the elastic balance task for two representative participants. The graph illustrates the sway path at three time points (PRE, POST, FU) for both ROB (blue) and TRAD (black). Participant S002 was selected for TRAD, while S004 was selected for ROB

*Elastic balance*. No significant main effect of time was found (all ps > 0.05). Interestingly, significant time*group interactions were observed for path length and trunk variability in EO condition (respectively, *p* = 0.047 and *p* = 0.017), revealing that participants in ROB group showed greater improvements compared to individuals in TRAD group (see Table 4; Fig. 2C). Post-hoc analyses revealed that PwMS in ROB showed a decreasing trend in path length between PRE and FU (*p* = 0.051; see Fig. 3 for a statokinesiogram of sway path from two representative PwMS), while trunk variability significantly decreased between PRE and FU (*p* = 0.030).

**Table 4:** Results from RM ANOVA on elastic balance outcomes

Parameter	Group	Time	Mean	SE	RM ANOVA, *p*
SA_eo	ROB	PRE	85.20	20.12	TIME, *p* = 0.615
		POST	45.76	18.56	TIME * GROUP, *p* = 0.495
		FU	59.19	19.92	
	TRAD	PRE	84.49	23.60	
		POST	87.37	21.77	
		FU	90.45	23.36	
SA_ec	ROB	PRE	271.45	50.43	TIME, *p* = 0.793
		POST	238.55	42.59	TIME * GROUP, *p* = 0.700
		FU	243.61	50.15	
	TRAD	PRE	314.96	59.14	
		POST	308.40	49.95	
		FU	345.72	58.81	
APO_eo	ROB	PRE	11.28	1.10	TIME, *p* = 0.764
		POST	9.71	1.03	TIME * GROUP, *p* = 0.159
		FU	10.19	0.99	
	TRAD	PRE	9.87	1.29	
		POST	11.09	1.21	
		FU	11.86	1.16	
MLO_eo	ROB	PRE	7.57	1.17	TIME, *p* = 0.550
		POST	5.99	0.84	TIME * GROUP, *p* = 0.773
		FU	6.35	1.19	
	TRAD	PRE	8.17	1.37	
		POST	7.71	0.98	
		FU	8.14	1.40	
APO_ec	ROB	PRE	19.39	1.78	TIME, *p* = 0.890
		POST	18.50	1.53	TIME * GROUP, *p* = 0.098
		FU	16.92	1.50	
	TRAD	PRE	19.26	2.09	
		POST	21.07	1.80	
		FU	21.69	1.76	
MLO_ec	ROB	PRE	14.74	1.74	TIME, *p* = 0.624
		POST	13.61	1.44	TIME * GROUP, *p* = 0.172
		FU	13.24	1.86	
	TRAD	PRE	15.01	2.04	
		POST	15.69	1.68	
		FU	18.52	2.18	
Path_length_eo	ROB	PRE	139.81	18.46	TIME, *p* = 0.934
		POST	116.16	15.96	TIME * GROUP, *p* = 0.047
		FU	108.75	12.75	
	TRAD	PRE	97.43	21.65	
		POST	131.02	18.72	
		FU	132.30	14.95	
Path_length_ec	ROB	PRE	228.38	28.62	TIME, *p* = 0.411
		POST	234.25	23.94	TIME * GROUP, *p* = 0.244
		FU	219.34	26.61	
	TRAD	PRE	233.41	33.56	
		POST	267.47	28.07	
		FU	283.39	31.20	
Romberg	ROB	PRE	0.35	0.70	TIME, *p* = 0.395
		POST	0.46	0.36	TIME * GROUP, *p* = 0.166
		FU	0.51	0.17	
	TRAD	PRE	1.61	0.82	
		POST	1.05	0.42	
		FU	0.48	0.20	
Trunk_var_eo	ROB	PRE	0.11	0.01	TIME, *p* = 0.408
		POST	0.09	0.01	TIME * GROUP, *p* = 0.017
		FU	0.08	0.01	
	TRAD	PRE	0.08	0.01	
		POST	0.11	0.02	
		FU	0.10	0.01	
Trunk_var_ec	ROB	PRE	0.19	0.02	TIME, *p* = 0.516
		POST	0.17	0.02	TIME * GROUP, *p* = 0.669
		FU	0.16	0.02	
	TRAD	PRE	0.20	0.03	
		POST	0.21	0.03	
		FU	0.20	0.02	

*Reactive balance*. One participant in TRAD did not complete the session due to fatigability (ROB *N* = 22; TRAD *N* = 15). Main effect of time was observed for first oscillation time (i.e., the average time it takes for the patient’s torso to stabilize after the first perturbation) in both frontal (Mean ± SE PRE: 1.94 ± 0.15; POST: 1.35 ± 0.13; FU: 1.37 ± 0.12; *p* = 0.004) and right (PRE: 1.71 ± 0.19; POST: 1.24 ± 0.14; FU: 1.14 ± 0.10; *p* = 0.021) directions, and average oscillation time (i.e., the average time it takes for the patient’s torso to stabilize) in frontal (PRE: 1.48 ± 0.11; POST: 1.14 ± 0.12; FU: 1.01 ± 0.06; *p* = 0.003) and right (PRE: 1.23 ± 0.12; POST: 0.86 ± 0.07; FU: 0.94 ± 0.07; *p* = 0.014) directions. In addition, time*group interactions were observed for first oscillation time in left and right (*p* = 0.027 and *p* = 0.029) directions, and for average oscillation time in both left and right (*p* = 0.006 and *p* = 0.012) directions, suggesting that individuals in ROB group showed a higher improvement compared to TRAD participants (see Table 5; Fig. 2B). Post-hoc analyses revealed that PwMS in ROB had greatest enhancement between PRE and POST (*p* = 0.024) and PRE and FU (*p* = 0.003) for first oscillation time in left, and across all time points for first oscillation time in right (PRE vs. POST: *p* = 0.024; PRE vs. FU; *p* < 0.001; POST vs. FU; *p* = 0.043). For average oscillation time in left, the significant differences in ROB group were observed across PRE and POST and PRE and FU (PRE vs. POST: *p* = 0.017; PRE vs. FU: *p* < 0.001). The same trend has been found for average oscillation time in right (PRE vs. POST: *p* = 0.008; PRE vs. FU; *p* < 0.001).

*Stability Limits.* No significant main effect of time or time*group were found across variables considered for this test (all ps > 0.05) (See Table 2 in Additional file 1).

**Table 5:** Results from RM ANOVA on reactive balance outcomes from hunova^®^

Parameter	Group	Time	Mean	SE	RM ANOVA, *p*	
First_osc_time_front (sec)	ROB	PRE	2.07	0.20	*TIME*, *p* = 0.004	
		POST	1.39	0.17	TIME * GROUP, *p* = 0.188	
		FU	1.16	0.15		
	TRAD	PRE	1.80	0.24		
		POST	1.31	0.21		
		FU	1.58	0.18		
First_osc_time_left (s)	ROB	PRE	1.89	0.25	TIME, *p* = 0.071	
		POST	1.21	0.16	TIME * GROUP, *p* = 0.027	
		FU	1.01	0.17		
	TRAD	PRE	1.58	0.30		
		POST	1.28	0.19		
		FU	1.85	0.21		
First_osc_time_right (s)	ROB	PRE	2.04	0.24	*TIME*, *p* = 0.021	
		POST	1.34	0.17	TIME * GROUP, *p* = 0.029	
		FU	0.88	0.13		
	TRAD	PRE	1.39	0.29		
		POST	1.14	0.21		
		FU	1.40	0.16		
Osc_time_front (s)	ROB	PRE	1.60	0.14	*TIME*, *p* = 0.003	
		POST	1.19	0.15	TIME * GROUP, *p* = 0.223	
		FU	0.90	0.07		
	TRAD	PRE	1.35	0.17		
		POST	1.09	0.18		
		FU	1.12	0.09		
Osc_time_left (s)	ROB	PRE	1.45	0.18	TIME, *p* = 0.114	
		POST	0.92	0.11	TIME * GROUP, *p* = 0.006	
		FU	0.65	0.12		
	TRAD	PRE	1.01	0.21		
		POST	0.97	0.14		
		FU	1.24	0.15		
Osc_time_right (s)	ROB	PRE	1.35	0.15	*TIME*, *p* = 0.014	
		POST	0.87	0.09	TIME * GROUP, *p* = 0.012	
		FU	0.68	0.09		
	TRAD	PRE	1.10	0.18		
		POST	0.85	0.11		
		FU	1.20	0.11		

## Conclusion

Balance impairment is one of the most disabling symptom in MS and leads to a decrease in quality of life [30, 39]. To addresses this, selecting appropriate rehabilitation strategies and applying them regularly is crucial in mitigating these disability-inducing symptoms. Recently, an increasing use of technological devices has been observed in the rehabilitation of PwMS with the aim of decreasing symptoms, reducing treatment costs, and improve treatment adherence [40]. The present study demonstrated that a rehabilitative protocol that combines robotic-assisted training with conventional physiotherapy (ROB) is at least non-inferior to a protocol consisting solely of traditional balance training (TRAD) for PwMS. The primary outcome and almost all other clinical measurements demonstrated an improved balance ability after rehabilitation treatment in both groups. Nonetheless, some interesting differences between ROB and TRAD were observed in unstable balance conditions measured through hunova^®^. Additionally, these improvements were maintained for at least two months post-treatment, aligning with the principle of reversibility in exercise training and supported by long-term measurements. As indicated by Schlagheck and colleagues (2021) [41], although eleven studies in this review included follow-up measurements, they were inconclusive in assessing the reversibility of training effects because they either did not re-evaluate the primary outcome, as we did in our study. To our knowledge, this study is among the first to provide evidence that the hunova^®^ robotic platform may be complementary or alternative approach to conventional rehabilitation strategies for balance disorders in MS.

In line with a recent review that demonstrated the potentials of robotic balance training in orthopaedic and neurological settings [42], our results extended these findings by showing that participants who received a robotic training with hunova^®^ achieved statistically significant additional improvements in dynamic balance parameters [18, 43, 44]. This study provides further support about the potentials of a technological sensorimotor protocol in improving balance disorder for PwMS that were only partially investigated before.

Both groups benefited from rehabilitation, as measured by the primary outcome BBS, which is the most commonly used metric for balance assessment in robotic rehabilitation studies [45]. In addition, in line with previous findings [13, 14], COMP from SOT improved after a balance training. Furthermore, participants in both groups increased their walking distance as measured by the 2MWT, consistent with evidence suggesting that exercises strengthening core muscles and improving proprioception can enhance both balance and walking ability in PwMS [20, 46], where proprioception refers to the body’s internal sensory system that provides continuous information about joint position, muscle stretch, and movement, and is essential for maintaining balance and coordinated motor actions.

Perceived fatigue did not show a significant change in our study after balance rehabilitation, despite the improvements observed at a qualitative level for both groups (see Table 1 in Additional File). This aligns with findings from previous studies where robotic interventions did not significantly affect fatigue levels [47], while others found beneficial effect after a robotic and/or conventional treatment for balance [13, 40]. One possible explanation is that the duration or intensity of our balance training may not have been sufficient to induce measurable changes in perceived fatigue, as fatigue is a multifaceted symptom that might require longer or more intensive interventions to show significant improvements. Additionally, although groups were comparable at baseline for MFIS scores, before the intervention, only ROB participants had values above the cut-off of 38 (ROB Mean ± SE = 41.82 ± 13.98 vs. TRAD = 34.05 ± 19.37), which has been proposed to identify fatigued patients [48]. This heterogeneity might have influenced the results, potentially masking an effect of the intervention.

While there were no significant differences in static balance or stability limits between the ROB and TRAD groups, dynamic balance improvements were more pronounced in the ROB group, in line with previous research on robotic rehabilitation in stroke populations [32]. One possible explanation is that the intervention primarily targeted dynamic balance components rather than static postural control. The training protocols in both groups included unstable balance exercises, which are inherently more focused on improving postural adaptations in response to external perturbations rather than enhancing static postural stability. It is also worth noting that robotic platforms like hunova^®^ are specifically designed to simulate real-life scenarios through perturbation exercises that are more effective in enhancing dynamic balance.

Interestingly, participants in the ROB group exhibited additional improvements in dynamic balance tasks, Although both groups significantly decreased in the time of the first oscillation in frontal and right direction and in the average oscillation time in both frontal and right direction, only PwMS in ROB group showed a marked decreased in first oscillation time and in average oscillation time in both right and left directions, suggesting that reaction to ML perturbation improved significantly after a target robotic treatment with random perturbation. This finding is in line with evidence from studies on older adults at risk of falls, which have reported that those who can execute a quick step in response to an induced perturbation exhibit greater stability [49]. Dynamic balance disorders are more evident in the ML than in the AP direction [16], in accordance with the view that ML stabilization is critical for walking [3] and responsible for hip fractures [12].

Overall, significant reductions in trunk displacement and oscillation during dynamic exercises in ROB group indicated better trunk control in response to unstable conditions. These data are consistent with other findings showing improved trunk behavior in neurological patients treated with robotic-assistive devices [32, 43]. Given that exercises focused on trunk control are directly correlated with gait recovery and rehabilitation, this suggests that rehabilitative interventions should include trunk balance rehabilitation as an important component of treatment [50].

Furthermore, comparing the groups, ROB showed a greater improvement in path length, in line with Castelli and colleagues (2023) [8], which found that, after a rehabilitation training delivered with hunova^®^, neurological population decreased their path length in dynamic balance condition, suggesting that robotic-assisted training can target specific components of postural control that are difficult to assess and train with conventional methods, as the use of foam or wooden balance boards [17]. While traditional methods do not simulate dynamic perturbations or provide detailed assessments [16], robotic systems as hunova^®^ offer continuous feedback and the opportunity to engage in more precise and task-specific training that promotes neuroplasticity in neurological patients [51].

In discussing our data, some important limitations need to be considered. Results may not generalize to people with a higher disability level who require walking aids such as a pair of canes or crutches (EDSS = 6.5) or with progressive MS. The follow-up period of two months post-intervention may not fully capture the long-term effects of the robotic or traditional rehabilitation protocols. A longer-term follow-up would be useful to assess the sustainability of the improvements. Some participants may have been unfamiliar with the robotic platform, which could have led to a learning curve or initial discomfort with the technology. This may have affected the initial results, especially if some participants took longer to adapt. In addition, future studies should consider incorporating dual-task exercises into robotic training protocols, as simultaneous cognitive and postural tasks have been shown to increase fall risk in PwMS [52, 53].

Another limitation of the present study is that we did not include neurophysiological or neuroimaging measures to directly assess neuroplastic changes after the training. Indeed, as pointed out in a recent systematic review by [54], advanced MRI markers (e.g., functional connectivity, diffusion metrics) are rarely incorporated in rehabilitation trials despite their potential to reveal adaptive structural and functional brain changes in PwMS. In future work, incorporating such MRI-based neuroplasticity markers would be highly valuable to elucidate whether our robotic intervention yields greater neuroplastic reorganization compared to conventional balance training.

Even though several technological rehabilitation approaches have proven to be effective for PwMS, the heterogeneity inherent to MS complicates the definition of universally accepted guidelines for developing standardized protocols for robotic training and conventional therapy in terms of frequency, intensity, duration, and type of training. So, it is important to understand the magnitude of mobility and balance improvement gained by a specific therapeutic dose, defined as “the amount of active ingredient(s) expected to produce the desired effect and the frequency and duration at which the agent is taken” [55]. Understanding the optimal dose of a given therapeutic intervention is critical to the implementation of evidence-based practice and to increase the effectiveness of interventions. For example, a meta-analysis involving 21 articles with 822 individuals with stroke [56] recommended effective protocols for balance and walking with 40–60-minute sessions, 2–5 times per week, over 8–12 weeks. Similar endorsements have been made in MS, emphasizing the importance of customizing protocols based on patients’ needs and severity of their symptoms. A review with 71 studies (N PwMS = 3306) [20] proposed that an appropriate dose for rehabilitation intervention should involve 4–12 weeks of treatment, with a frequency of 2–3 times per week and an average session duration of 40 min. While another review of 19 studies with 858 PwMS [51] found that the most effective dose for improving dynamic balance was between 8 and 19 weeks, with sessions held twice a week, lasting 20–30 min each. While our study aligns with previous recommendations (i.e., 2 sessions per week of 45 min each, for a total of 10 weeks), future research should further investigate the optimal therapeutic dose in a fully robotic-centered protocol using the hunova^®^, rather than in protocols where it is only partially integrated with traditional physiotherapy. Nevertheless, our results, which demonstrate the non-inferiority of the robotic protocol for traditional measures and additional improvements in fine-grained dynamic balance metrics, emphasize the importance of a multimodal rehabilitation approach. Combining different training modalities within MS care pathways could provide significant benefits and facilitate the integration of advanced technologies alongside conventional therapies.

## Supplementary Information

Supplementary Material 1

## Data Availability

The datasets used and analyzed during the current study are available from the corresponding author on reasonable request.

## Abbreviations

2MWT: Two-Minute walking test
APO: Anterior–posterior oscillation
AP: Anterior–posterior
BBS: Berg balance scale
CoG: Center of gravity
COMP: Composite score of sensory organization test
CoP: Center of pressure
CNS: Central nervous system
EC: Eyes-closed
EDSS: Expanded disability status scale
EO: Eyes-open
IMU: Inertial movement unit
M: Mean
MFIS_COG: Cognitive subscale of the Modified fatigue impact scale
MFIS_PHYS: Physical subscale of the Modified fatigue impact scale
MFIS_PSY-SOC: Psycosocial subscale of the Modified fatigue impact scale
MFIS_TOT: Total score of the Modified fatigue impact scale
ML: Mediolateral
MLO: Medio-lateral oscillation
MoCA: Montreal cognitive assessment
MS: Multiple sclerosis
N: Number
PwMS: People with multiple sclerosis
RR: Relapsing-remitting
SA: Sway area
SD: Standard deviation
SDMT: Symbol digit modalities test
SE: Standard error
sec: Seconds
SOT: Sensory organization test
TUG: Timed-up-and-go
