# Supplementary material for: Improving dynamic balance in relapsing-remitting MS: insights from robotic-assisted rehabilitation therapy
Source: J Neuroeng Rehabil. 2026 Jan 9;23:58. doi: 10.1186/s12984-025-01856-w (PMC12882523; doi:10.1186/s12984-025-01856-w)
Supplement: Supplementary file 1 — Supplementary Material 1 [file 12984_2025_1856_MOESM1_ESM.docx]

**Additional file 1**

**Table 1 – Results from RM ANOVA on static balance variables from hunova®**

| **Parameter** | **Group** | **Time** | **Mean** | **SE** | **RM ANOVA, p** | |  |
| --- | --- | --- | --- | --- | --- | --- | --- |
| SA_eo | ROB | PRE | 6.19 | 1.64 | TIME, p = 0.105 | |  |
|  |  | POST | 6.51 | 1.67 | TIME * GROUP, p = 0.231 | | |
|  |  | FU | 7.06 | 3.38 |  |  |  |
|  | TRAD | PRE | 7.06 | 1.92 |  |  |  |
|  |  | POST | 8.04 | 1.96 |  |  |  |
|  |  | FU | 12.74 | 3.96 |  |  |  |
| SA_ec | ROB | PRE | 24.40 | 5.47 | TIME, p = 0.445 | |  |
|  |  | POST | 26.45 | 6.08 | TIME * GROUP, p = 0.269 | | |
|  |  | FU | 22.33 | 9.86 |  |  |  |
|  | TRAD | PRE | 30.26 | 6.42 |  |  |  |
|  |  | POST | 20.74 | 7.12 |  |  |  |
|  |  | FU | 41.43 | 11.56 |  |  |  |
| APO_range_eo | ROB | PRE | 3.15 | 0.31 | TIME, p = 0.197 | |  |
|  |  | POST | 3.14 | 0.39 | TIME * GROUP, p = 0.508 | | |
|  |  | FU | 3.40 | 0.42 |  |  |  |
|  | TRAD | PRE | 3.13 | 0.36 |  |  |  |
|  |  | POST | 3.75 | 0.46 |  |  |  |
|  |  | FU | 3.89 | 0.50 |  |  |  |
| MLO_range_eo | ROB | PRE | 2.42 | 0.34 | TIME, p = 0.359 | |  |
|  |  | POST | 2.28 | 0.26 | TIME * GROUP, p = 0.302 | | |
|  |  | FU | 2.39 | 0.37 |  |  |  |
|  | TRAD | PRE | 2.42 | 0.40 |  |  |  |
|  |  | POST | 2.56 | 0.31 |  |  |  |
|  |  | FU | 3.01 | 0.44 |  |  |  |
| APO_range_ec | ROB | PRE | 6.51 | 0.82 | TIME, p = 0.636 | |  |
|  |  | POST | 6.57 | 0.64 | TIME * GROUP, p = 0.341 | | |
|  |  | FU | 5.51 | 0.63 |  |  |  |
|  | TRAD | PRE | 6.63 | 0.96 |  |  |  |
|  |  | POST | 5.83 | 0.75 |  |  |  |
|  |  | FU | 6.97 | 0.73 |  |  |  |
| MLO_range_ec | ROB | PRE | 5.04 | 0.57 | TIME, p = 0.906 | |  |
|  |  | POST | 5.33 | 0.72 | TIME * GROUP, p = 0.129 | | |
|  |  | FU | 4.17 | 0.71 |  |  |  |
|  | TRAD | PRE | 4.95 | 0.67 |  |  |  |
|  |  | POST | 4.67 | 0.85 |  |  |  |
|  |  | FU | 5.70 | 0.84 |  |  |  |
| Path_lenght_eo | ROB | PRE | 51.91 | 4.80 | TIME, p = 0.731 | |  |
|  |  | POST | 50.48 | 5.15 | TIME * GROUP, p = 0.430 | | |
|  |  | FU | 47.18 | 4.74 |  |  |  |
|  | TRAD | PRE | 45.68 | 5.63 |  |  |  |
|  |  | POST | 47.69 | 6.03 |  |  |  |
|  |  | FU | 47.55 | 5.56 |  |  |  |
| Path_lenght_ec | ROB | PRE | 110.39 | 12.31 | TIME, p = 0.472 | |  |
|  |  | POST | 103.88 | 10.02 | TIME * GROUP, p = 0.133 | | |
|  |  | FU | 87.20 | 9.63 |  |  |  |
|  | TRAD | PRE | 102.58 | 14.43 |  |  |  |
|  |  | POST | 93.91 | 11.75 |  |  |  |
|  |  | FU | 110.89 | 11.29 |  |  |  |
| Romberg | ROB | PRE | 0.38 | 0.19 | TIME, p = 0.759 | |  |
|  |  | POST | 0.42 | 0.08 | TIME * GROUP, p = 0.060 | | |
|  |  | FU | 0.79 | 0.17 |  |  |  |
|  | TRAD | PRE | 0.71 | 0.22 |  |  |  |
|  |  | POST | 0.45 | 0.09 |  |  |  |
|  |  | FU | 0.41 | 0.19 |  |  |  |

**Table 2 – Results from RM ANOVA on stability limits variables from hunova®**

| **Parameter** | **Group** | **Time** | **Mean** | **SE** | **RM ANOVA, p** |
| --- | --- | --- | --- | --- | --- |
| SA | ROB | PRE | 101.38 | 5.06 | TIME, p = 0.725 |
|  |  | POST | 100.85 | 4.21 | TIME * GROUP, p = 0.778 |
|  |  | FU | 101.04 | 4.15 |  |
|  | TRAD | PRE | 108.22 | 5.94 |  |
|  |  | POST | 105.70 | 4.94 |  |
|  |  | FU | 109.80 | 4.87 |  |
| Max_cop_back | ROB | PRE | 5.63 | .19 | TIME, p = 0.611 |
|  |  | POST | 5.77 | .20 | TIME * GROUP, p = 0.502 |
|  |  | FU | 5.63 | .18 |  |
|  | TRAD | PRE | 5.88 | .23 |  |
|  |  | POST | 5.68 | .24 |  |
|  |  | FU | 5.59 | .21 |  |
| Max_cop_front | ROB | PRE | 7.75 | .31 | TIME, p = 0.377 |
|  |  | POST | 7.66 | .16 | TIME * GROUP, p = 0.820 |
|  |  | FU | 7.87 | .23 |  |
|  | TRAD | PRE | 8.19 | .36 |  |
|  |  | POST | 8.21 | .18 |  |
|  |  | FU | 8.61 | .27 |  |
| Max_cop_left | ROB | PRE | 7.80 | .27 | TIME, p = 0.846 |
|  |  | POST | 7.60 | .22 | TIME * GROUP, p = 0.534 |
|  |  | FU | 7.58 | .27 |  |
|  | TRAD | PRE | 7.58 | .32 |  |
|  |  | POST | 7.85 | .26 |  |
|  |  | FU | 7.64 | .31 |  |
| Max_cop_right | ROB | PRE | 7.17 | .24 | TIME, p = 0.237 |
|  |  | POST | 7.24 | .25 | TIME * GROUP, p = 0.697 |
|  |  | FU | 7.41 | .22 |  |
|  | TRAD | PRE | 7.48 | .29 |  |
|  |  | POST | 7.28 | .29 |  |
|  |  | FU | 7.68 | .26 |  |
